# Improving the metabolic fidelity of cancer models with a physiological cell culture medium

**DOI:** 10.1126/sciadv.aau7314

**Published:** 2019-01-02

**Authors:** Johan Vande Voorde, Tobias Ackermann, Nadja Pfetzer, David Sumpton, Gillian Mackay, Gabriela Kalna, Colin Nixon, Karen Blyth, Eyal Gottlieb, Saverio Tardito

**Affiliations:** 1 Cancer Research UK Beatson Institute, Garscube Estate, Switchback Road, Glasgow, G611BD, UK.; 2 Institute of Cancer Sciences, University of Glasgow, Glasgow, UK.; 3 Technion Integrated Cancer Center, Faculty of Medicine, Technion (Israel Institute of Technology), Haifa, Israel.

## Abstract

Currently available cell culture media may not reproduce the in vivo metabolic environment of tumors. To demonstrate this, we compared the effects of a new physiological medium, Plasmax, with commercial media. We prove that the disproportionate nutrient composition of commercial media imposes metabolic artifacts on cancer cells. Their supraphysiological concentrations of pyruvate stabilize hypoxia-inducible factor 1α in normoxia, thereby inducing a pseudohypoxic transcriptional program. In addition, their arginine concentrations reverse the urea cycle reaction catalyzed by argininosuccinate lyase, an effect not observed in vivo, and prevented by Plasmax in vitro. The capacity of cancer cells to form colonies in commercial media was impaired by lipid peroxidation and ferroptosis and was rescued by selenium present in Plasmax. Last, an untargeted metabolic comparison revealed that breast cancer spheroids grown in Plasmax approximate the metabolic profile of mammary tumors better. In conclusion, a physiological medium improves the metabolic fidelity and biological relevance of in vitro cancer models.

## Teaser

Plasmax, a physiological cell culture medium, prevents metabolic artifacts imposed on cancer cells by commonly used media.

## INTRODUCTION

It seems obvious that the nutrient composition of the culture medium affects the phenotypic behavior of cells, their response to stresses and stimuli, epigenotype, and transcriptome. However, until recently (*1*–*5*), very little attention has been paid to perfecting the formulation of historic media. The vast majority of biomedical researchers use cell culture media that were not designed to reproduce the physiological cellular environment but were formulated to enable the continued culture of cells with minimal amounts of nutrients and serum (i.e., Eagle’s minimal essential medium). Later, these media were modified by multiplying the concentration of selected nutrients, such as glucose and glutamine, by a factor of 4, to avoid nutrient exhaustion when leaving the culture unattended for longer time periods [i.e., Dulbecco’s modified Eagle’s medium (DMEM)]. Therefore, it is not surprising that several recent reports highlight discrepancies between in vitro and in vivo cancer cell metabolism (*6*, *7*). We have previously designed a medium containing amino acids, glucose, and pyruvate at physiological concentrations (*2*, *3*). More recently, a culture medium was formulated on the basis of the composition of human blood, and its use proved to profoundly affect in vitro cancer cell metabolism (*1*).

Aiming to investigate and reduce the gap between in vitro observations and tumor biology, we implemented a complex culture medium, herein called Plasmax, composed of more than 50 nutrients and metabolites at the concentration normally found in human blood, and we directly compared its effects on cultured cells against standard commercial media. This new medium includes metabolites with marginal nutritional value, but whose physiological relevance to cellular metabolism cannot be discounted. This is exemplified by uric acid, the end product of purine catabolism, which inhibits pyrimidine synthesis, a pathway central for cancer cell anabolism (*1*). In this study, we compare triple-negative breast cancer (TNBC) cell lines cultured in Plasmax or in DMEM-F12, a commercially available, nutrient-rich medium. We show that the adoption of a more physiological culture medium, which includes the micronutrient selenium, substantially influences the colony-forming capacity of cancer cells by preventing ferroptosis. This form of cell death caused by iron-dependent lipid peroxidation is counteracted by selenoproteins, such as glutathione peroxidase 4 (GPX4), whose expression and activity are dependent on selenium availability (*8*, *9*). Furthermore, the transcriptional and metabolic phenotypes of cells cultured in Plasmax are reprogrammed in a way that is largely independent of the cell proliferation rate. This suggests that the supraphysiological concentrations of nutrients (e.g., glucose and glutamine) present in most commercial media are not essential to sustain cell proliferation and misshape the metabolism of cancer cells. This aspect was studied in depth by relating the availability of nutrients in the media to their exchange rates and intracellular levels. Last, by means of an untargeted metabolomics approach, we compared the profiles of breast cancer cells cultured in Plasmax and DMEM-F12 with orthotopic xenografts grown in mice, providing evidence that Plasmax more closely recapitulates the metabolic environment of tumors.

## RESULTS

### The formulation of a physiological medium: Plasmax

To design a chemically defined medium with nutrients and metabolites at concentrations normally found in the blood of healthy human individuals, we consulted a publicly available database (www.serummetabolome.ca). On the basis of the normal concentration range reported for human blood (meeting the arbitrary threshold of 2 μM), commercial availability, and chemical stability, we selected proteinogenic and nonproteinogenic amino acids, amino acid derivatives, and other components as reported in table S1. These compounds were dissolved in stock solutions and added to Earle’s balanced salt solution (EBSS), together with salts, vitamins, and trace elements, to obtain a ready-to-use cell culture medium, herein called Plasmax (see Materials and Methods and table S1 for details on formulation and stock solutions). Despite having been designed and developed independently, the composition of Plasmax is similar to the recently reported human plasma-like medium (HPLM) (table S1) (*1*), thus cross-validating the methods used to formulate these physiological media. To minimize the influence of components contributed by the bovine serum, all the experiments were performed by supplementing the media with 2.5% fetal bovine serum (FBS) unless otherwise indicated.

**Fig. 1:**
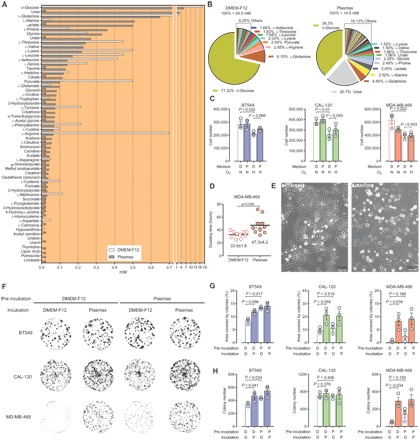
Plasmax sustains cancer cell growth and increases colony formation. (**A** and **B**) Comparison of the formulation of Plasmax and DMEM-F12. na (not applicable) refers to components not present in DMEM-F12, with the exception of linoleate, putrescine, lipoic acid, and thymidine, which are not present in Plasmax. (**C**) Cell number at the end of a proliferation assay with BT549, CAL-120, and MDA-MB-468 cells, performed in Plasmax or DMEM-F12 under normoxic (N) and hypoxic (H) conditions, 21 and 0.1% O_2_, respectively. Means ± SEM; *n* = 3 independent experiments. (**D**) Doubling time of MDA-MB-468 cells as determined after each of the 10 consecutive passages in either DMEM-F12 or Plasmax. Means ± SEM. (**E**) Micrographs showing the morphology of MDA-MB-468 cells cultured in DMEM-F12 or Plasmax for eight passages. (**F**) Representative images and (**G** and **H**) quantification of a colony formation assay performed with BT549, CAL-120, and MDA-MB-468 cells preincubated (2 days), seeded at 500 cells per well, and incubated (12 days) with DMEM-F12 (D) or Plasmax (P) as indicated. Means ± SEM (*n* = 3 independent experiments). (C, D, G, and H) Each dot represents an independent experiment, and *P* values refer to a two-tailed *t* test for paired homoscedastic samples.

To compare the effect of different culture media on the phenotypic behavior of cells, we selected DMEM-F12, a commercially available medium that is richer than MEM and DMEM in terms of nutrient composition and is suitable for growth of a wide range of cell types even under low-serum conditions. The direct comparison of the formulation of Plasmax and DMEM-F12 is reported in Fig. 1A. Of note, while glucose and glutamine together account for almost three-quarters of the entire pool of nutrients in DMEM-F12 (24.5 mM), these components contribute less than half of the whole pool in Plasmax (14.5 mM, Fig. 1B). Moreover, 35% of the total nutrient availability within Plasmax is made up of components that are not present in DMEM-F12, thereby providing more nutritional options for the cells.

### Plasmax sustains breast cancer cell growth in vitro and increases colony formation capacity

We tested the effects of Plasmax and DMEM-F12 on the human TNBC cell lines BT549, CAL-120, and MDA-MB-468 that were routinely cultured in DMEM-F12 supplemented with 10% FBS. Cells were preincubated for at least 48 hours in the respective media, both containing 2.5% FBS, to allow adaptation to the experimental conditions. To prevent exhaustion of the nutrients that are avidly consumed by cancer cells and present at lower concentrations in Plasmax than in DMEM-F12 (e.g., glucose and glutamine), the ratio between the volume of medium and number of cells was maintained in excess of 1 ml/100,000 cells/day.

In normoxia, all cell lines proliferated at comparable rates in the two media, with only the MDA-MB-468 cells showing a trend toward slower proliferation in Plasmax (Fig. 1C). To investigate this further, we cultured MDA-MB-468 cells for several passages in the two media. Their doubling time was calculated at each passage and demonstrated a 1.5-fold slower growth rate in Plasmax compared to DMEM-F12 (Fig. 1D). Alongside the effect on proliferation, Plasmax affected the morphology of MDA-MB-468 cells. Phase contrast microscopy indicated that, when cultured in Plasmax, MDA-MB-468 cells were flatter, and the associations between neighboring cells appeared to be looser than when these cells were grown in DMEM-F12 (Fig. 1E). In addition, when cells were plated at low density, Plasmax increased the colony formation capacity of all cell lines, with the most pronounced effect being observed in MDA-MB-468 cells (Fig. 1, F to H). Consistently, a 2-day preincubation with Plasmax was sufficient to slightly increase the number of colonies formed by BT549 and MDA-MB-468 cells when incubated in DMEM-F12 (Fig. 1H). These results show that the effect of Plasmax on cells persists following its withdrawal, suggesting the accumulation of a medium component or the triggering of signaling pathways advantageous for colony formation.

### Identification of sodium selenite as the Plasmax component enhancing the colony formation capacity of TNBC cells

**Fig. 2:**
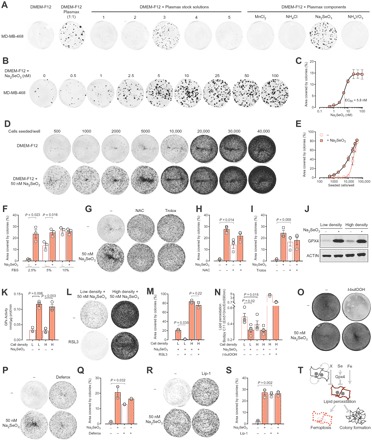
The Plasmax component sodium selenite prevents ferroptosis and thereby enhances the colony formation capacity of TNBC cells. (**A**) Images of colony formation assays performed with MDA-MB-468 cells seeded 500 cells per well and incubated for 14 days in 1:1 DMEM-F12/Plasmax mix or in DMEM-F12 supplemented with the different mixed stock solutions or individual components of Plasmax. Images are representative of three independent experiments. (**B**) Representative images and (**C**) quantification of colony formation assays performed with MDA-MB-468 cells seeded at 500 cells per well in DMEM-F12 with different concentrations of Na_2_SeO_3_ and incubated for 14 days. Means ± SEM; *n* = 3 independent experiments. (**D**) Representative images and (**E**) quantification of the colony formation assays performed with MDA-MB-468 cells seeded at the indicated cell density and incubated for 7 days. Means ± SEM; *n* = 3 independent experiments. (**F**) Quantification of colony formation assays performed with MDA-MB-468 cells seeded at 5000 cells per well and incubated for 7 days in DMEM-F12 supplemented with FBS and 50 nM Na_2_SeO_3_, as indicated. Means ± SEM; *n* = 3 independent experiments. (**G**) Representative images and (**H** and **I**) quantification of colony formation assays performed with MDA-MB-468 cells seeded at 5000 cells per well and incubated for 7 days in DMEM-F12 with 50 nM Na_2_SeO_3_, 1 mM *N*-acetylcysteine (NAC), or 100 μM Trolox, as indicated. Means ± SEM; *n* = 3 independent experiments. (**J**) Western blot showing GPX4 levels in MDA-MB-468 cells seeded at low (2000 cells/cm^2^) or high (10,000 cells/cm^2^) density and incubated in DMEM-F12 for 3 days with 50 nM Na_2_SeO_3_, as indicated. Images are representative of three independent experiments. (**K**) GPX activity measured in MDA-MB-468 cells seeded and incubated as for (J). Means ± SEM; *n* = 3 independent experiments. (**L**) Representative images and (**M**) quantification of colony formation assays performed with MDA-MB-468 cells seeded at 5000 cells per well (L) or at 50,000 cells per well (H) and incubated for 7 days in DMEM-F12 supplemented with 50 nM Na_2_SeO_3_ and 250 nM (1*S*,3*R*)-RSL3. Means ± SEM; *n* = 2 independent experiments. (**N**) Lipid peroxidation levels of MDA-MB-468 cells seeded as in (J) and incubated in DMEM-F12 for 48 hours with 50 nM Na_2_SeO_3_ and for the last hour with 50 μM *t*-butOOH, as indicated. At the end of the incubation, 1 μM BODIPY 581/591 C11 was added to the cells for 15 min. Means ± SEM; *n* = 3 or 4 independent experiments. (**O**) Representative images of MDA-MB-468 cells seeded at 10,000 cells/cm^2^ and incubated in DMEM-F12 with Na_2_SeO_3_ for 72 hours and for the last 24 hours with 50 μM *t*-butOOH, as indicated. Images are representative of three independent experiments. (**P** and **R**) Representative images and (**Q** and **S**) quantification of colony formation assays performed with MDA-MB-468 cells seeded at 5000 cells per well and incubated for 7 days in DMEM-F12 supplemented with 50 nM Na_2_SeO_3_, 2.5 μM deferoxamine, and 50 nM liproxstatin-1 (Lip-1), as indicated. Means ± SEM; *n* = 2 (P and Q) or *n* = 3 (R and S) independent experiments. (**T**) Schematic representation of the factors affecting the colony-forming capacity of TNBC cells. “X” refers to a factor present in the medium of confluent MDA-MB-468 cells and not yet identified. (F, H, I, K, M, N, Q, and S) Each dot represents an independent experiment, and *P* values refer to a two-tailed *t* test for paired homoscedastic samples.

To assess whether Plasmax increased or DMEM-F12 suppressed the colony formation capacity, we incubated MDA-MB-468 cells in a 1:1 mixture of both media (Fig. 2A). Since this resulted in a colony number comparable to that obtained in Plasmax, we further investigated which component of Plasmax was responsible for stimulating colony formation. By systematically adding the mixed stock solutions (table S1) and, subsequently, the individual components of Plasmax to DMEM-F12, we identified sodium selenite as the component of Plasmax, which was sufficient to increase colony formation in MDA-MB-468 (Fig. 2A), BT549, and CAL-120 cells (fig. S1). The colony-stimulating activity of selenite was dose dependent, with the maximal effect observed at ~25 nM (Fig. 2, B and C), and evident when the FBS supplementation was lower than 10% (Fig. 2F). In line with the results obtained by comparing the effects of Plasmax and DMEM-F12 (Fig. 1, C and F to H), selenite promoted survival and proliferation of cells seeded at low density, while its effect progressively diminished with the increase of the number of cells seeded (Fig. 2, D and E). Selenium is incorporated into the active site of selenoproteins as selenocysteine, and hence, it modulates the antioxidant capacity of cells. We therefore tested the effects of antioxidants such as *N*-acetylcysteine and the vitamin E derivative, Trolox. Both compounds significantly rescued the area covered by colonies in the absence of selenium supplementation, proving that oxidative stress hinders colony formation (Fig. 2, G to I). Glutathione peroxidases (GPXs) are antioxidant enzymes whose activity largely depends on the presence of selenium in their active site. In particular, since GPX4 is regulated by selenium availability (*8*), we tested the effects of selenite supplementation on GPX4 levels in MDA-MB-468 cells. Independent of the cell density, the expression (Fig. 2J) and activity (Fig. 2K) of GPX4 were markedly increased in the presence of selenite. Next, to investigate the role of GPX4 in the colony-forming process, we seeded cells at low and high densities and incubated them with selenite and (1*S*,3*R*)-RSL3, a specific GPX4 inhibitor. Despite selenite supplementation, this compound significantly decreased the capacity of cells to form colonies when seeded at low density without affecting high-density cell cultures (Fig. 2, L and M). This suggested that high GPX4 activity is required to maintain the redox balance in isolated cells but is dispensable in more confluent cultures. We then measured the level of peroxidized lipids, a preferential substrate for GPX4, using the fluorescent probe BODIPY C11 581/591. Lipid peroxidation was significantly higher in cells seeded at low cell density in the absence of selenite compared to similar cell cultures supplemented with selenite (Fig. 2N). Moreover, selenium-deprived cells showed significantly higher levels of lipid peroxidation when seeded at low cell density compared to high-density cell cultures (Fig. 2N). These results demonstrate that selenium supplementation prevents lipid peroxidation by maintaining high levels of GPX4 activity, and thereby increases the fitness of breast cancer cells to form colonies. Notably, more confluent cells also require selenium for maintaining their redox balance and viability if challenged with a peroxidizing agent such as *tert*-butylhydroperoxide (*t*-butOOH; Fig. 2, N and O). Since GPX4 inhibition and lipid peroxidation have been shown to cause ferroptosis (*9*), we assessed whether this type of iron-dependent cell death could explain the defect in colony formation of selenium-deprived cells. Therefore, a colony-forming assay was performed by seeding cells at low density in the presence of an iron chelator, deferoxamine, or a ferroptosis inhibitor, liproxstatin-1. The results show that the colony-forming capacity in the absence of selenite was significantly increased by deferoxamine and completely rescued by liproxstatin-1 (Fig. 2, P to S). Together, we demonstrated that breast cancer cells seeded at low density in the absence of selenium undergo cytotoxic lipid peroxidation, resulting in ferroptotic cell death (Fig. 2T). In addition, we elucidated the mechanism through which a cell culture medium composition can significantly affect the results obtained in assays that are commonly used in biomedical research.

### Plasmax normalizes the pseudohypoxic state of cells cultured in commercial media

**Fig. 3:**
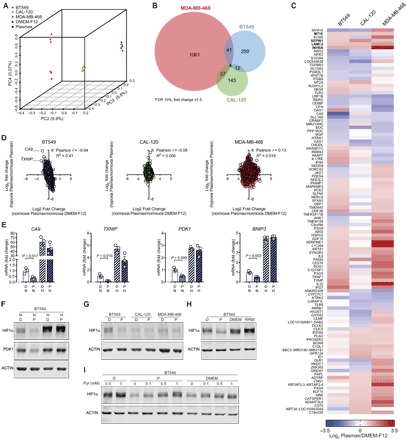
Plasmax induces cell line–specific transcriptomic alterations and prevents the pseudohypoxic gene expression signature of cells cultured in commercial media. (**A**) PCA of gene expression obtained from RNA-seq data of BT549, CAL-120, and MDA-MB-468 cells cultured in Plasmax or DMEM-F12, in normoxia. *n* = 3. Each symbol represents an independent experiment. (**B**) Venn diagram showing the number of genes that were differentially regulated in these cell lines when cultured in Plasmax and DMEM-F12. (**C**) Heat map of genes that were significantly [false discovery rate (FDR) of 10%] and coherently regulated (absolute log_2_ fold change ≥ 0.585) by culturing cells in Plasmax, in normoxia, in at least two of three cell lines. *n* = 3, log_2_ (mean fold change). (**D**) Correlation analysis of genes regulated by hypoxia in Plasmax (*y* axis) and genes regulated by Plasmax in normoxia (*x* axis). Each dot represents the mean of three independent experiments. (**E**) Expression levels of HIF1α target genes *CA9*, *TXNIP*, *PDK1*, and *BNIP3* in BT549 cells relative to control condition (normoxia, DMEM-F12). Means ± SEM; *n* = 3, each dot represents an independent experiment, and *P* values refer to a two-tailed *t* test for unpaired homoscedastic samples. (**F**) Western blot showing HIF1α levels in BT549 cells in Plasmax (P) or DMEM-F12 (D), in normoxia (N) and hypoxia (H). (**G**) Western blot showing HIF1α levels in BT549, CAL-120, and MDA-MB-468 cells cultured in DMEM-F12 or Plasmax in normoxia. (**H**) Western blot showing HIF1α levels in BT549 cells cultured in normoxia in DMEM-F12, Plasmax, DMEM, and RPMI 1640. (**I**) Western blot showing pyruvate-dependent HIF1α levels in BT549 cells, cultured in DMEM-F12, Plasmax, and DMEM in normoxia. (F to I) Images are representative of three independent experiments. *P* values refer to a two-tailed *t* test for unpaired homoscedastic samples.

To investigate the effects of the medium composition on gene expression, we cultured BT549, CAL-120, and MDA-MB-468 cells in Plasmax or DMEM-F12 in the presence of 21% or 0.1% O_2_ (normoxia and hypoxia, respectively), and we evaluated the transcriptional response by RNA sequencing (RNA-seq). First, the RNA profiles in normoxia were compared by a principal components analysis (PCA). While components 1 to 3 mainly separated the individual cell lines, component 4 separated the cells according to the culture media (Fig. 3A and fig. S2). In particular, more than a thousand genes were differentially regulated by the two media in MDA-MB-468 cells (Fig. 3B). However, of all the genes that were significantly regulated by the media in at least one cell line, only 5% of genes were coordinately regulated in two or more cell lines, and only four genes (*INHBA*, *SEPW1*, *LAMC2*, and *MT1E*) were coordinately regulated in all three cell lines (Fig. 3, B and C). This indicates that the broad transcriptional response induced by a different nutritional environment is cell line specific, even within a group of cells that originate from the same cancer subtype (i.e., TNBC).

Subsequently, the effect of the culture media on the response to hypoxia was investigated. Hypoxia reduced the proliferation of all cell lines. However, Plasmax provided a minor growth advantage to hypoxic BT549 and CAL-120 cells (Fig. 1C). In addition, in BT549 cells, the transcriptional response to hypoxia was medium dependent. A correlation analysis demonstrated that genes that were significantly induced in hypoxia in BT549 cells were down-regulated by Plasmax (compared to DMEM-F12) under normoxic conditions (Fig. 3D). This indicated a hypoxic gene expression signature in cells cultured in DMEM-F12, even under normoxia. Several genes following this inverse correlation are established hypoxia-inducible factor 1α (HIF1α) targets, such as *CA9*, *TXNIP*, *PDK1*, and *BNIP3*. While the expression of these genes was induced by hypoxia independently of the media, their expression levels under normoxic conditions were significantly decreased in Plasmax compared to DMEM-F12 (Fig. 3E). Consistent with this regulatory pattern, the protein levels of HIF1α and its target PDK1 under normoxia were reduced in BT549 cells when they were cultured in Plasmax (Fig. 3F). Of note, the levels of normoxic HIF1α were comparable in all three cell lines when cultured in Plasmax (Fig. 3G). To test whether the effect on the normoxic stabilization of HIF1α was specific to DMEM-F12, we incubated BT549 cells in two other commonly used commercial media (i.e., DMEM and RPMI 1640). Both media increased the normoxic levels of HIF1α even further (Fig. 3H). These results demonstrate that nonphysiological media can impose a chronic pseudohypoxic state and that this is avoided by culturing cells in Plasmax.

Given the large number of differences between the composition of DMEM-F12 and Plasmax, a systematic elimination/addition approach was used, and pyruvate was identified as the factor regulating HIF1α in normoxia. The levels of HIF1α in BT549 cells incubated in Plasmax, DMEM-F12, DMEM, and RPMI 1640 positively correlated with the pyruvate concentrations supplemented in those media (0.1, 0.5, 1, and 1 mM, respectively; Fig. 3H). Furthermore, pyruvate was sufficient to induce HIF1α levels in a dose-dependent manner in different media (Fig. 3I). Together, these results show that supraphysiological concentrations of pyruvate, which are commonly found in commercial media, do not affect the response of cells to oxygen limitation, but can lead to the stabilization of HIF1α, inducing a pseudohypoxic response in normoxia.

### The metabolism of cancer cells is skewed by the availability of nutrients in the medium

**Fig. 4:**
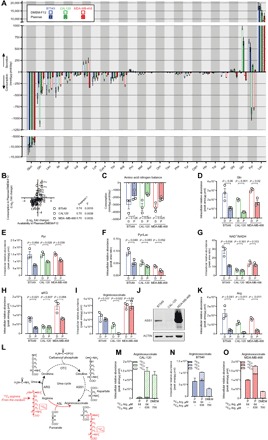
Culture medium defines cell metabolic landscape by altering nutrient exchange rates and metabolic pathways. (**A**) Consumption and secretion rates of amino acids and intermediates of glycolysis and the urea cycle in cells cultured in DMEM-F12 and Plasmax. Means ± SEM; *n* = 3 independent experiments. (**B**) Correlation analysis between the differential availability of neutral proteinogenic amino acids in Plasmax compared to DMEM-F12 and their respective consumption rates. Means ± SEM; *n* = 3 independent experiments. *P* values refer to a two-tailed Pearson test. (**C**) Consumption of the amino acid bound nitrogen. Means ± SEM; *n* = 3 independent experiments. (**D** to **I** and **K**) Intracellular abundance of metabolites and metabolite ratios in cells cultured in Plasmax and DMEM-F12. Means ± SEM; *n* = 3 independent experiments. (**J**) Western blot showing ASS1 levels in cells cultured in DMEM-F12. Images are representative of three independent experiments. (**L**) Schematic representation of the urea cycle and expected labeling of argininosuccinate from ^13^C_6_ arginine upon reversed ASL activity. (**M** to **O**) Intracellular levels of ^13^C argininosuccinate in CAL-120 (M), BT549 (N), and MDA-MB-468 (O) cells cultured for 48 hours in Plasmax and DMEM supplemented with ^13^C_6_ and ^13^C_0_ arginine, as indicated. Means ± SEM; *n* = 3 (M) or *n* = 2 (N and O) independent experiments. *P* values refer to a two-tailed *t* test for paired homoscedastic samples.

Next, we compared the effects of Plasmax and DMEM-F12 on cell metabolism. First, we determined the exchange rates of nutrients and metabolites between cells and media (Fig. 4A). For both media, glucose was the most consumed nutrient, and lactate was the most abundantly released metabolite. The exchange rates of glucose and lactate were not significantly different in the two media. Since the concentration of glucose is 3-fold higher in DMEM-F12 than in Plasmax (17.5 and 5.56 mM, respectively), we can conclude that, under these conditions, the rate of glucose fermentation is independent of its extracellular availability. In contrast, the consumption of glutamine was affected by the media. All three cell lines consumed markedly less glutamine in Plasmax, where it is supplemented at 0.65 mM, compared to DMEM-F12, which contains 2 mM glutamine. We also found a correlation between availability and consumption for nutrients other than glutamine. For all cell lines, a significant, positive correlation was found between the medium concentrations of the neutral proteinogenic amino acids and the rate at which they were consumed and/or secreted (Fig. 4B). In particular, all cell lines released alanine in DMEM-F12 but consumed it in Plasmax, where it is 10-fold more concentrated (0.05 mM versus 0.51 mM). Since alanine is central for the transfer of nitrogen between amino acids (i.e., transamination), we tested whether the balance of aminoacidic nitrogen was affected by the media (Fig. 4C). The net consumption of amino acid–derived nitrogen was higher in DMEM-F12 compared to Plasmax, showing that, in DMEM-F12, the release of alanine does not compensate for the increased metabolism of other amino acids. In line with this, the intracellular concentration of glutamine, another major nitrogen carrier, was elevated in DMEM-F12 compared to Plasmax in the three cell lines (Fig. 4D). Together, these results show that supplementing media with concentrations of amino acids that deviate from those found in serum causes proportional changes in their uptake/release and metabolism. Moreover, these changes occur without pronounced effects on proliferation (Fig. 1C), indicating that an increase in amino acid consumption does not predict its essentiality for growth.

All cell lines showed decreased intracellular levels of pyruvate when cultured in Plasmax compared with DMEM-F12 (Fig. 4E). As shown in Fig. 3 (G to I), the supraphysiological pyruvate concentration of commercial media stabilizes HIF1α, specifically in BT549 cells. In line with this, BT549 cells were also distinct from the other two lines in terms of pyruvate metabolism. While CAL-120 and MDA-MB-468 cells consumed pyruvate from DMEM-F12 (where it is supplemented at 500 μM), and released it in Plasmax (where it is present at 100 μM), BT549 cells exported large quantities of pyruvate in both media (Fig. 4A). In addition, BT549 cells cultured in DMEM-F12 showed high pyruvate/lactate and NAD^+^/NADH ratios, which were both normalized by Plasmax to those observed in CAL-120 and MDA-MB-468 cells (Fig. 4, F and G). Unexpectedly, the pyruvate-dependent stabilization of HIF1α in BT549 cells cultured in DMEM-F12 occurred despite significantly elevated levels of α-ketoglutarate, an obligate substrate of the prolyl-hydroxylases that promotes the degradation of HIF1α (Fig. 4H).

The medium-dependent effects on metabolic processes are cell line specific, supporting the view that the nutritional environment rewires cell metabolism based on the (epi)genome. This was further exemplified when studying arginine metabolism. Argininosuccinate is an intermediate of the urea cycle produced by argininosuccinate synthetase 1 (ASS1) from citrulline and aspartate and was found to be decreased in several cancer cell lines when cultured in a physiological culture medium (*1*). The intracellular levels of argininosuccinate in CAL-120 cells were substantial in DMEM-F12 but close to the detection limit when cultured in Plasmax (Fig. 4I). Since the gene encoding argininosuccinate synthetase 1, *ASS1*, undergoes epigenetic silencing in multiple types of cancer (*10*), we tested its protein expression, which was barely detectable in CAL-120 cells (Fig. 4J). These results indicate that the levels of ASS1 and its product, argininosuccinate, were uncoupled in these cells when cultured in DMEM-F12. Arginine is 10-fold more abundant in DMEM-F12 (0.699 mM) than in Plasmax (0.064 mM), and the higher extracellular concentration translates into increased intracellular arginine levels (Fig. 4K). While argininosuccinate lyase (ASL) produces arginine and fumarate from argininosuccinate in the urea cycle, we hypothesized that the supraphysiological levels of arginine present in DMEM-F12 could reverse the reaction catalyzed by ASL and produce argininosuccinate directly from arginine in ASS1-deficient cells (Fig. 4L). This hypothesis was tested by incubating CAL-120 cells in Plasmax or DMEM (commercially available without arginine) supplemented with ^13^C_6_ arginine to reach a concentration of 0.7 mM. This increased the intracellular levels of ^13^C_6_ arginine (fig. S3A) and argininosuccinate (Fig. 4M). Confirming our hypothesis, the ^13^C_6_ isotopologue of argininosuccinate accounted for more than 95% of its total intracellular pool, while less than 0.5% was derived from citrulline via the canonical urea cycle, where one of the six carbons of arginine is lost to produce urea (^13^C_5_ argininosuccinate; Fig. 4M). These results demonstrate that ASL activity is reversed under these conditions and explain the increased levels of argininosuccinate in CAL-120 cells cultured in DMEM-F12. To test whether this metabolic anomaly was specific to ASS1-deficient cells, ^13^C_6_ arginine was also traced in ASS1-proficient cell lines (BT549 and MDA-MB-468). The isotopologue distribution of argininosuccinate demonstrates that, upon exposure to supraphysiological levels of arginine, ASL activity is reversed in those cells as well (Fig. 4, N and O). Whereas the presence of ^13^C_5_ ornithine indicated arginase (ARG) activity in all three cell lines (fig. S3B), ^13^C_5_ citrulline was not detected under any condition (fig. S3C), indicating that either the mitochondrial carbamoyl phosphate synthase 1 (CPS1) or ornithine transcarbamylase (OTC) is not active. Consistently, also in ASS1-proficient cells cultured in DMEM supplemented with ^13^C_6_ arginine, the entire argininosuccinate pool consisted of ^13^C_6_ argininosuccinate (Fig. 4, N and O). However, when cultured in Plasmax, most of the argininosuccinate was derived from citrulline uptaken from the medium (^13^C_0_ argininosuccinate; Fig. 4, N and O). This observation suggests that, under physiological conditions, these ASS1-proficient cells depend on the exogenous availability of citrulline for the production of argininosuccinate. Overall, we show that concentrations of arginine commonly present in cell culture media reverse a reaction of the urea cycle in TNBC cells and thereby mask the metabolic hallmarks of ASS1-deficient cells. Moreover, our findings highlight that, in the absence of exogenous citrulline (i.e., in commercial media), ASS1-proficient breast cancer cells also produce argininosuccinate mainly via reversed ASL activity.

### Cells cultured in Plasmax better recapitulate the metabolic signature of tumors

**Fig. 5:**
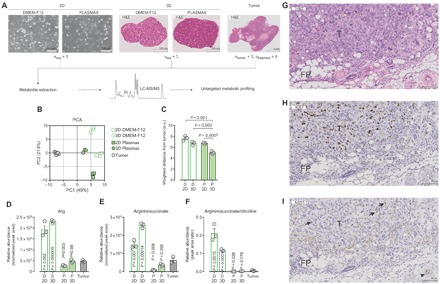
Cells cultured in Plasmax approximate the in vivo tumor metabolome better. (**A**) Schematic representation of the experimental setup applied to compare the untargeted metabolic profiling of CAL-120 cells cultured in vitro (as 2D monolayers or 3D spheroids) in Plasmax and DMEM-F12, with CAL-120–derived orthotopic tumors. (**B**) PCA of three independent experiments or *n* = 3 mice (nine tumor fragments) performed as detailed in (A). (**C**) Weighted distance between each culture condition and the mean values of tumor samples, as calculated from the PCA reported in (B). *P* values refer to a two-tailed *t* test for unpaired homoscedastic samples. a.u., arbitrary units. (**D** to **F**) Relative abundance and ratio of urea cycle intermediates in CAL-120 cells grown in Plasmax and DMEM-F12 as 2D and 3D cultures and orthotopic mammary tumors. Means ± SEM; *n* = 3 independent experiments or *n* = 3 mice. *P* values refer to a two-tailed *t* test for unpaired homoscedastic samples comparing the in vitro conditions to the tumor samples. (**G** to **I**) Micrographs of CAL-120–derived mammary tumor tissue showing (G) hematoxylin and eosin (H&E) staining and IHC for (H) Ki67 and (I) ASS1. Arrows indicate stromal cells, whereas the arrowhead denotes vessel endothelium. T, tumor; FP, fat pad.

Next, we compared the metabolic profiles of CAL-120 cells cultured in vitro, in DMEM-F12 and Plasmax, with the profile of CAL-120–derived mammary tumors. For this purpose, CAL-120 cells were grown as an adherent monolayer [two-dimensional (2D)] and in suspension as spheroids (3D) in the two different media. To obtain orthotopic tumors, we implanted CAL-120 cells, cultured in DMEM-F12 as monolayers, in the mammary fat pad of mice (Fig. 5A). The liquid chromatography tandem mass spectrometry (LC-MS/MS) results were analyzed by means of an untargeted metabolomics approach, which allowed the comparison of samples with different biological matrices. By combining the ions detected in both positive and negative modes, this analytical approach identified ~100 compounds that were analyzed with a PCA (Fig. 5B). The PCA grouped the samples according to the biological conditions. For both 2D and 3D cultures, cells grown in Plasmax were clearly separated from cells grown in DMEM-F12. Tumor samples clustered away from the in vitro conditions, showing that the variation between the in vivo and in vitro metabolic profiles exceeds the variation observed between tumors grown in different animals. Of the in vitro conditions, cells cultured in 3D in Plasmax clustered closest to the tumor (Fig. 5, B and C), indicating that, by adopting a 3D culture method and physiological medium, the overall metabolic phenotype of tumors can be better approximated.

As shown in Fig. 4 (L to O), supraphysiological concentrations of arginine cause the reversal of one of the reactions of the urea cycle in vitro. This resulted in the accumulation of argininosuccinate in ASS1-deficient cells cultured in DMEM-F12 but not in Plasmax (Fig. 4, I and J). To test whether this rewiring of the urea cycle was relevant in vivo, we assessed the levels of intermediates of the urea cycle in tumors and cultured cells. Arginine levels were comparable between tumors and cells cultured in Plasmax, but were ~4-fold higher in cells cultured in DMEM-F12 (Fig. 5D). As a consequence of the different arginine availability, the level of argininosuccinate was higher in cells cultured in DMEM-F12 compared with tumors (Fig. 5E). The levels of argininosuccinate were found to be lower in cells cultured in Plasmax than in tumors, a difference that may be explained by the intratumoral presence of ASS1-proficient stromal cells. An immunohistochemistry (IHC) analysis confirmed the ASS1 deficiency of cancer cells and showed ASS1-positive stromal cells and vascular endothelium (*11*) in tumor tissue (Fig. 5, G to I). In addition, the argininosuccinate/citrulline ratio, a further readout of ASS1 activity, was markedly higher in cells cultured in DMEM-F12 compared with the tumor or with cells cultured in Plasmax (Fig. 5F). Together, these results demonstrate that the reversed activity of ASL, caused by the supraphysiological concentration of arginine in commercial media, is an artifactual phenotype that is not relevant to tumor biology and that can be avoided by using a culture medium based on physiology such as Plasmax.

## DISCUSSION

Current cancer research heavily relies on results obtained using cell culture. Addressing the many discrepancies between in vivo biology and cell-based models (e.g., nonphysiological media composition, lack of waste product removal, and the often hyperoxic environment) may improve the reliability of results obtained in vitro. This study objectively shows that efforts to refine culture conditions improve the robustness and fidelity of in vitro cancer cell models. It demonstrates that the culture medium formulation significantly affects the results obtained in commonly used cell biology assays, such as those testing colony formation and gene expression. Plasmax is a newly formulated medium composed of nutrients and metabolites at the concentration normally found in human plasma. Here, we provided the composition and procedure to produce Plasmax as a resource to address specific physiopathological questions more accurately. The use of this, as well as another medium independently formulated on a similar rationale (*1*), could help to explain some of the metabolic discrepancies between results obtained with in vitro cell culture and in vivo models (*4*, *6*, *7*). The present study and Cantor *et al*. (*1*) show that the disproportionate and nonphysiological concentrations of nutrients typically found in commercial media skew the metabolism of cancer cells at both the intracellular and exchange flux levels (Fig. 4) and generate undesired artifactual phenotypes (Figs. 3 to 5). These insights should be considered when designing studies that aim to identify the dependency of cancer cells on specific nutrients or metabolic pathways (i.e., metabolic addictions) or when investigating the effects of antimetabolites.

To provide proof of concept that the use of physiological media can also unveil novel biological findings, we mechanistically elucidated the causes underlying phenotypic, transcriptomic, and metabolic differences found in cells cultured in Plasmax and DMEM-F12. First, we revealed a novel role for sodium selenite as a stimulator of the colony formation capacity of breast cancer cells (Fig. 2). Selenium, one of the essential mineral nutrients typically contributed to culture media by FBS, is supplemented to Plasmax as sodium selenite and is incorporated in selenoproteins such as GPXs. The expression of GPX4 was increased upon selenite supplementation, and its high activity prevented lipid peroxidation. Ferroptosis, a type of cell death caused by iron-dependent lipid peroxidation, was identified as the cause of the compromised colony formation capacity of cells cultured in DMEM-F12 with reduced FBS and was prevented by antioxidants, iron chelators, and selenite. While the potential anticancer and cancer-preventive effects of selenium supplementation have been the subject of many studies (*12*), our results indicate that sodium selenite may provide a cell-autonomous survival advantage to isolated cancer cells in a hostile environment. Of note, selenite (or Plasmax) did not confer any survival or growth advantage when cells were cultured as more confluent cultures (Figs. 1C and 2, D and E). We found that the conditioned medium of MDA-MB-468 cells cultured at high density rescued the colony-forming capacity of selenium-deprived cells, suggesting that cells secrete a yet unidentified, protective factor. The translational potential of our findings is substantiated by the increased incidence of metastases observed in a mouse model of breast cancer upon dietary supplementation of sodium selenite (*13*) and calls for further investigation.

Next, we show that pyruvate, at the concentrations present in DMEM-F12 and other standard media, induces a pseudohypoxic response mediated by HIF1α stabilization. Different from previous reports (*14*, *15*), the HIF1α stabilization observed in 21% oxygen was independent of the rate of aerobic glycolysis, which is comparable in Plasmax and DMEM-F12 (Fig. 4A), but dependent on the concentration of exogenous pyruvate (Fig. 3I), which may affect the activity of prolyl hydroxylases that regulate the stability of HIF1α (*15*).

In addition, we discovered that concentrations of arginine typically found in commercial media (~0.7 mM) reverse the activity of the urea cycle enzyme ASL in the three TNBC cell lines analyzed in this work (Fig. 4, L to O). Tracing supraphysiological concentrations of ^13^C_6_ arginine showed that the urea cycle is truncated in these cell lines at the level of the mitochondrial reactions (OTC or CPS1) and at the ASS1-catalyzed reaction (CAL-120). The reversal of the ASL-catalyzed reaction has been previously reported in fumarate hydratase-deficient cells, where the high intracellular concentration of fumarate favors the synthesis of argininosuccinate from arginine (*16*). While, in such context, the reversed activity of ASL could have a physiopathological justification, the same reaction triggered by the excessive arginine supplementation has no relevance to tumor biology. Our findings indicate that, under physiological conditions, ASS1-proficient TNBC cells mainly use exogenous citrulline, and not arginine or arginine-derived ornithine, for the production of argininosuccinate (Fig. 4, M to O, and fig. S3). In ASS1-deficient cells, excessive arginine availability masks their distinctive metabolic traits: impaired coupling of citrulline to aspartate and consequent low argininosuccinate levels (Fig. 4I).

However, comparing only in vitro conditions prevents unambiguous conclusions on their relevance to tumor biology. Therefore, we compared the metabolism of CAL-120 breast cancer cells grown in vitro as adherent monolayers or as anchorage-independent spheroids, in both DMEM-F12 and Plasmax, to an orthotopic mouse model of breast cancer (Fig. 5). While previous reports showed that anchorage withdrawal is sufficient to rewire the tricarboxylic acid cycle of cultured cancer cells (*17*) or identified metabolic vulnerabilities of cancer cells using advanced culture systems (*18*), this is, to our knowledge, the first attempt to directly compare the general metabolic profiles of cells cultured in different media with an in vivo cancer model. Although the relative comparison of metabolite levels measured in different biological matrices (i.e., cultured cells versus tumors) requires careful normalization and should be cautiously interpreted, the levels of argininosuccinate indicated that these ASS1-deficient cells do not reverse the ASL-catalyzed reaction in vivo, as they do when cultured in nonphysiological media (Fig. 5E). This is corroborated by the argininosuccinate/citrulline ratio, a normalization-independent index of ASS1 activity (Fig. 5F). Together, this provides proof of principle that the use of a physiological medium, such as Plasmax, can prevent in vitro artifacts that would otherwise require experimental work with laboratory animals to be disproved.

While specific metabolic parameters of tumors can be better approximated by either 3D culture conditions [e.g., citrate metabolism (*17*)] or use of a physiological medium (e.g., urea cycle reversal, as shown here), their combination represents a better model of the overall metabolic profile of tumor cells. We can speculate that the remaining gap between tumors and in vitro conditions could be further bridged by taking into account the metabolic differences between murine and human blood, the composition of interstitial fluid, and the organ- and stroma-specific (*19*) metabolic environment. However, a robust untargeted metabolic comparison between mammary tumors grown in mice, and cancer cells cultured under different in vitro conditions, proved that a culture medium based on the composition of human blood is a significant improvement compared to current standards.

In conclusion, we have shown that a physiologically formulated medium can provide new insights into the role of trace elements in cancer biology, prevents artifacts imposed by the unbalanced nutritional composition of conventional media, improves the degree to which in vitro models reflect the metabolism of tumors, and may offer opportunities for reduced use of animal models in cancer biology.

## MATERIALS AND METHODS

### Plasmax production

The components of Plasmax are reported in table S1. Stock solutions were prepared in individual tubes for single use and stored at −78°C. To increase the solubility of its components, the pH of solution 1 was adjusted to 1.00 with HCl, and the pH of solution 4 (urate) was adjusted to 13.30 with NaOH. To prepare a bottle of Plasmax, stock solutions 1 to 5 were added to 500 ml of commercially available EBSS (cat. no. 24010043; Thermo Fisher Scientific, MA, USA), in combination with l-glutamine (cat. no. 25030081; Thermo Fisher Scientific), sodium pyruvate (S8636; Sigma-Aldrich, MO, USA), and 2.5% FBS (Thermo Fisher Scientific). After preparation and equilibration with 5% CO_2_, the pH of different batches of Plasmax was 7.1 to 7.3, with thus not needing for further adjustment. The buffering capacity of Plasmax ensured that its pH remained stable when MDA-MB-468 cells (seeded at 200,000 cells per well) were cultured in 8 ml of medium for 72 hours. The osmolarity of Plasmax was 280 mOsm/kg as measured with a vapor pressure osmometer (Wescor 5500). Trace elements (including sodium selenite; table S1) were supplemented at the concentrations found in Advanced DMEM-F12 (cat. no. 12634028; Thermo Fisher Scientific).

### Cell culture

The following human TNBC cell lines were used: BT549 and MDA-MB-468 (both obtained from A. Schulze, Biocenter, Wurzburg, Germany, and originally purchased from the American Type Culture Collection, VA, USA) and CAL-120 (DSMZ, Braunschweig, Germany). All cell lines were authenticated using the Promega GenePrint 10 Kit (Promega, WI, USA) and tested negative for mycoplasma infection using the MycoAlert Mycoplasma Detection Kit (Lonza, Bazel, Switzerland). Before performing experiments to compare different media, cells were maintained in DMEM-F12 (cat. no. 21331046; Thermo Fisher Scientific) supplemented with 2 mM glutamine (Thermo Fisher Scientific) and 10% FBS (Thermo Fisher Scientific). Cells were cultured at 37°C and 5% CO_2_. To determine the doubling time, MDA-MB-468 cells were cultured in DMEM-F12 and Plasmax supplemented with 2.5% FBS for at least 10 consecutive passages. At each passage, the cells were trypsinized and counted, and the same number of cells (500,000 or 1,000,000) were seeded in a 10-cm dish with the respective media and allowed to grow until 80% confluent. For the identification of the Plasmax component responsible for HIF1α stabilization, BT549 cells were preincubated in DMEM-F12 or Plasmax (both supplemented with 2.5% FBS) for 2 days and subsequently (day 2) seeded in six-well plates at 50,000 cells per well in 2 ml of the respective culture medium. After 24 hours (day 3), culture medium was changed to 8 ml of medium (DMEM-F12, DMEM, or RPMI 1640 for cells preincubated in DMEM-F12; Plasmax for cells preincubated in Plasmax) with different concentrations of pyruvate. Cells were allowed to proliferate for 72 hours, after which cell lysates were prepared (day 6) for immunoblotting.

### Immunoblotting

Cells were lysed in Laemmli sample buffer (Bio-Rad, CA, USA) supplemented with 2-mercaptoethanol. Lysates were heated at 95°C for 5 min and loaded on 10% gels (Bio-Rad) for SDS–polyacrylamide gel electrophoresis. After electrophoretic separation, proteins were transferred onto 0.2 μm nitrocellulose membranes (Amersham, Germany), blocked with 5% nonfat milk [in tris-buffered saline (TBS) + 0.01% Tween], and incubated overnight at 4°C with the following primary antibodies: ACTIN (1:500; SC1616; Santa Cruz, TX, USA), ASS1 (D4O4B) XP (1:1000; cat. no. 70720; Cell Signaling, MA, USA), GPX4 (1:1000; ab125066, Abcam, Cambridge, UK), HIF1α (1:500; cat. no. 610959, BD Biosciences, CA, USA), and PDK1 (1:1000; MSP49; MitoSciences, OR, USA). Membranes were washed and incubated with near-infrared fluorescent secondary antibodies (1 hour at room temperature; 1:5000; LI-COR, NE, USA). Proteins were detected with the LI-COR Odyssey Imaging System.

### Immunohistochemistry

Tissue sections (4 μm) were cut from formalin-fixed, paraffin-embedded blocks. These sections were baked onto poly-lysine slides at 60°C before one set was stained with H&E to investigate morphology and check suitability for further investigation. The following antibodies were used for IHC: Ki67 (RM-9106; Thermo Fisher Scientific) and ASS1 (cat. no. 70720; Cell Signaling). The sections were deparaffinized in xylene, rehydrated through graded ethanols, and washed in deionized water before undergoing heat-induced epitope retrieval using a Dako pretreatment module. All sections were heated for 25 min at 98°C in 10 mM sodium citrate (pH 6) retrieval buffer (TA-250-PM1X, Thermo Fisher Scientific). Sections were washed in TBS with Tween (TBST). The staining took place on a Dako Autostainer Link 48. The antibodies against Ki67 and ASS1 were used at 1:100 and 1:1000 dilutions, respectively. Sections were then incubated with Dako EnVision rabbit secondary antibody, washed with TBST, and stained with Dako liquid diaminobenzidine. The sections were counterstained with hematoxylin, taken through graded alcohols, xylene, and then a glass coverslip applied with DPX mountant for microscopy (CellPath). Slides were scanned with a Leica SCN400F slide scanner (Leica Biosystems, Wetzlar, Germany), and images were acquired through the Leica Digital Image Hub.

### In vivo models

All in vivo experiments were carried out in dedicated barriered facilities proactive in environmental enrichment under the EU Directive 2010 and Animal (Scientific Procedures) Act 1986 (Home Office Project License number: 60-4181) with ethical review approval (University of Glasgow). Animals were cared for by trained and licensed individuals.

### Statistical analysis

Unless explicitly stated, three independent biological experiments were performed. Statistical analyses were performed with GraphPad Prism 7 using two-tailed, homoscedastic Student’s *t* tests for unpaired or paired samples as indicated in the figure legends or a Pearson correlation test as shown in Figs. 3D and 4B. Statistical analysis of RNA-seq was performed as described below.

### RNA sequencing

BT549, CAL-120, and MDA-MB-468 cells were preincubated in DMEM-F12 or Plasmax (both supplemented with 2.5% FBS) for 2 days. Subsequently (day 2), cells were seeded in six-well plates at 50,000 cells per well (BT549 and CAL-120) or 100,000 cells per well (MDA-MB-468) in 2 ml of the respective culture medium. After 24 hours (day 3), culture medium was changed to 8 ml of the respective medium, and cells were further incubated for 72 hours under atmospheric (21% O_2_, normoxia) or hypoxic (0.1% O_2_, hypoxia) conditions. On day 6, cell number was determined using a Coulter counter (*n* = 3 wells per condition), and cells were harvested for RNA extraction [*n* = 1 well per condition; samples were processed using the QIAshredder/RNeasy Mini Kit (Qiagen)].

For RNA-seq, samples were processed with on-column deoxyribonuclease digestion and diluted to 1.5 μg of RNA per 50 μl of water, and the quality of the purified RNA was tested on an Agilent 2200 TapeStation using RNA screentape. Libraries for cluster generation and DNA sequencing were prepared following an adapted method from Fisher *et al*. (*20*) using Illumina TruSeq RNA Library Preparation Kit v2. Quality and quantity of the DNA libraries were assessed on an Agilent 2200 TapeStation (D1000 screentape) and Qubit (Thermo Fisher Scientific), respectively. The libraries were run on the Illumina NextSeq 500 using the High Output 75 cycles kit (2 × 36 cycles, paired-end reads, single index). Quality control of the raw RNA-seq data files was performed by FastQC (www.bioinformatics.babraham.ac.uk/projects/fastqc/) and fastq_screen (www.bioinformatics.babraham.ac.uk/projects/fastq_screen/). Then, RNA-seq reads were aligned to the human genome (GRCh38.75) using TopHat2 (*21*), and resulting bam files were processed with htseq_count (https://htseq.readthedocs.io/en/release_0.10.0/). The final counts were normalized and analyzed with DESeq2 (*22*). Statistically significant differences in gene expression were determined with an FDR of 10%. In the PCA, the first principal component explains 85.1% of the variance. The second, third, and fourth components explain 8.9, 5.8, and 0.1% of the variance, respectively.

### Colony formation assays

BT549, CAL-120, and MDA-MB-468 cells were preincubated in DMEM-F12 or Plasmax (both supplemented with 2.5% FBS) for 2 days for the experiments reported in Fig. 1, and for all experiments, cells were seeded in six-well plates in 2 ml of medium. Cell densities, incubation times, and components added to the media are indicated in Figs. 1 and 2. Fresh medium was added after 96 hours to prevent nutrient exhaustion. At endpoint, colonies were fixed with trichloroacetic acid (final concentration of 3%, 30 min incubation at room temperature), washed with water, and stained with sulforhodamine B (0.057% in 1% acetic acid, 1 hour incubation at room temperature). LI-COR Odyssey and Image Studio 5 were used for image acquisition, and ImageJ was used for quantification of area covered by colonies and colony number. For the identification of sodium selenite as a stimulator of colony formation, MDA-MB-468 cells were cultured in a mixture of DMEM-F12 and Plasmax (1:1) and in DMEM-F12 supplemented with the different Plasmax stock solutions or individual Plasmax components reported in table S1.

### Lipid peroxidation assay

MDA-MB-468 cells were seeded at low (2000 cells/cm^2^) or high (10,000 cells/cm^2^) cell density in DMEM-F12 in the presence or absence of 50 nM sodium selenite. After a 48-hour incubation, cells were incubated with 1 μM BODIPY 581/591 C11 (Thermo Fisher Scientific) for 15 min. Cells were washed three times with phosphate-buffered saline (PBS), trypsinized, and resuspended in PBS with EDTA (ethylenediaminetetraacetic acid) and DAPI (4′,6-diamidino-2-phenylindole) before fluorescence-activated cell sorting (FACS) analysis (excitation, 488 nm). Lipid peroxidation was assayed in 10,000 events by the change in fluorescence and represented as the ratio Em510nm/Em590nm. As a positive control for lipid peroxidation, cells seeded at high cell density were exposed to 50 μM *t*-butOOH for 1 hour and analyzed by FACS, as described, or incubated for an additional 23 hours to assay the effect on cell survival.

### GPX expression and activity analysis

MDA-MB-468 cells were seeded at low (2000 cells/cm^2^) or high (10,000 cells/cm^2^) cell density in DMEM-F12 in the presence or absence of 50 nM sodium selenite. After a 72-hour incubation, cell lysates were prepared in radioimmunoprecipitation assay buffer for immunoblotting as described above, or GPX activity was analyzed using the Glutathione Peroxidase Assay Kit (ab102530; Abcam) as per the manufacturer’s instructions.

### Targeted metabolic analysis

BT549, CAL-120, and MDA-MB-468 cells were cultured in DMEM-F12 or Plasmax, and exchange rates/intracellular concentrations of nutrients and metabolites were determined. Cells were preincubated in DMEM-F12 or Plasmax (both supplemented with 2.5% FBS) for 3 days. Subsequently, on day 3, cells were seeded in six-well plates at 100,000 cells per well (BT549 and CAL-120) or 200,000 cells per well (MDA-MB-468) in 2 ml of the respective culture medium. After 24 hours (day 4), 2 ml of culture medium was added to each well to prevent nutrient exhaustion. On day 5, culture medium was changed to 2 ml (for medium extractions to calculate exchange rates) or 7 ml (for cell extractions to determine intracellular metabolite concentrations). At this time point, parallel wells were harvested for protein determination (see μg prot day5 in the equation below). Different volumes of culture media per well allowed quantification of the exchange rates for metabolites with slow flux and, in parallel, prevented artifacts when measuring intracellular levels of avidly consumed metabolites. After 24 hours (day 6), medium and cell extracts were prepared. Medium extracts: Twenty microliters of culture medium (spent medium or concomitantly incubated cell-free medium) was added to 980 μl of a cold extraction solution (−20°C) composed of methanol, acetonitrile, and water (5:3:2). Cell extracts: Cells were rapidly washed three times with ice-cold PBS, after which intracellular metabolites were extracted with 600 μl of ice-cold extraction solution for 5 min at 4°C. Medium and cell extracts were centrifuged (10 min at 16,000*g*) to remove insoluble material, and the supernatant was collected for LC-MS analysis. To allow data normalization and estimation of secretion/consumption rate, protein concentrations were determined on days 5 and 6 using a modified Lowry protein assay.

The LC-MS was performed as described previously (*23*). Briefly, a Q Exactive Plus Orbitrap Mass Spectrometer (Thermo Fisher Scientific) was used with a resolution of 35,000 at 200 mass/charge ratio (*m*/*z*), electrospray ionization, and polarity switching mode to enable both positive and negative ions across a mass range of 75 to 1000 *m*/*z*, and it was coupled with a Thermo Ultimate 3000 high-performance liquid chromatography (HPLC) system. The HPLC setup consisted of a ZIC-pHILIC column (SeQuant; 150 mm by 2.1 mm, 5 μm; Merck KGaA, Darmstadt, Germany), with a ZIC-pHILIC guard column (SeQuant; 20 mm by 2.1 mm). Biological extracts were injected (5 μl), and compounds were separated with a mobile phase gradient of 15 min, which started with 20% ammonium carbonate [20 mM (pH 9.2)] and 80% acetonitrile, and terminated with 20% acetonitrile. Flow rate and column temperature were maintained at 200 μl/min and 45°C, respectively, for a total run time of 23 min. All metabolites were detected using lock masses and a mass accuracy below 5 ppm. Thermo Xcalibur was used for data acquisition, and LCquan or TraceFinder 3.2 was used for analysis. The peak areas of metabolites were determined by using the exact mass of the singly charged ions. The retention time of metabolites was predetermined on the pHILIC column by analyzing an in-house mass spectrometry metabolite library, which includes the IROA Sigma-Aldrich MSMLS. The exchange rate per day [secretion (+) or consumption (−)] for a specific metabolite (*x*) was obtained according to the following equation

$$x=\frac{\Delta \text{metabolite}}{(\mu g\;\text{prot day}\;5+\mu g\;\text{prot day}\;6)/2}$$

where Δmetabolite = ((*x*)nmol_spent medium_ − (*x*)nmol_cell-free medium_). The total consumption of amino acid–derived nitrogen was calculated by adding up the consumption of each individual amino acid multiplied by its number of nitrogen atoms.

### ^13^C_6_ arginine tracing

Cells were seeded in six-well plates at 100,000 cells per well (BT549 and CAL-120) or 200,000 cells per well (MDA-MB-468) in 2 ml of DMEM-F12 or Plasmax (both supplemented with 2.5% FBS). After 24 hours (day 1), culture medium was changed to 8 ml of DMEM containing 700 μM ^13^C_6_ arginine (Cambridge Isotope Laboratories, MA, USA) (cells seeded in DMEM-F12) or Plasmax (as such or supplemented to 700 μM Arg with ^13^C_6_ arginine; cells seeded in Plasmax). After 48 hours (day 3), cells were extracted in 400 μl of ice-cold extraction solution, and samples were analyzed by LC/MS as described above.

### Untargeted metabolic profiling of cell monolayers, 3D spheroids, and tumors

CAL-120 cells were grown as 2D monolayers, and cell extracts for metabolomics were prepared as described above. Cells were extracted in 400 μl of ice-cold extraction solution. For each of the three independent experiments, cell extracts of three wells per condition were combined, and the protein content was determined in three wells incubated in parallel. All cell extracts were normalized to 2.16 μl of extraction solution per microgram of protein before LC-MS/MS analysis.

CAL-120 cells were seeded in DMEM-F12 or Plasmax (both supplemented with 2.5% FBS) at 4 × 10^6^ cells per 200 ml of culture medium in glass spinner flasks (40 rev/min; cat. no. F7689; Techne; Cole-Parmer Ltd., UK) to allow sphere formation. After 72 hours (day 3), the cell cultures were supplemented with 100 ml of the respective medium to prevent nutrient exhaustion. On day 4, spheres were collected and embedded in paraffin for IHC or used for extraction of metabolites. Spheres were allowed to sediment, and culture medium volume was reduced to 1 ml. After a brief centrifugation step (1 min, 600*g*), the supernatant was removed, spheres were washed quickly with 1 ml of ice-cold PBS and centrifuged for 10 s, and the pellet was extracted in 1 ml of ice-cold extraction solution (vortexed briefly, and incubated for 5 min at 4°C). After centrifugation (10 min at 16,000*g*), the supernatant (cell extract) was collected for LC-MS/MS analysis, and the pellet was used to determine the protein content with a modified Lowry protein assay. Before LC-MS/MS analysis, cell extracts were diluted to 2.16 μl of extraction solution per microgram of protein, normalizing the samples to the extracts obtained from the 2D monolayers.

Female severe combined immunodeficient mice (*n* = 3; 7 to 8 weeks old; Charles River Laboratories) were injected in the mammary fat pad with 4 × 10^6^ CAL-120 cells (cell suspension in 50 μl of Matrigel). Tumors were allowed to establish for 160 days, after which mice were humanely euthanized using schedule 1 methods. Tumors were quickly resected, divided into fragments, and either fixed in 10% neutral-buffered formalin for IHC or flash frozen on dry ice for extraction of metabolites. Frozen tumor fragments (two to four fragments per tumor; nine fragments in total) were weighed and dissociated in cold extraction solution (40 mg/ml) using ceramic beads and a Precellys homogenizer (Bertin Instruments, Montigny-le-Bretonneux, France). After centrifugation (10 min, 16,000*g*), the supernatant was used for LC-MS/MS analysis.

Samples were analyzed with the same LC/MS setup described above. However, for this experiment, metabolites were separated over a 30-min mobile phase gradient, decreasing the acetonitrile content to 20%, at a flow rate of 100 μl/min. The total analysis time was 40 min, and metabolites were detected using the mass spectrometer at a resolution of 70,000 (at 200 *m*/*z*). A pooled sample comprising a mixture of all sample extracts was analyzed using the same HPLC conditions but running the mass spectrometer in single ionization mode to acquire the positive and negative ions in separate runs and using data-dependent fragmentation (ddMS2) to improve the confidence in metabolite identification. Data were analyzed using Compound Discoverer software (Thermo Scientific v2.1). Retention times were aligned across all sample data files (maximum shift, 3 min; mass tolerance, 5 ppm). Unknown compound detection (minimum peak intensity of 3 × 10^6^) and grouping of compound adducts were carried out across all samples (mass tolerance, 5 ppm; retention time tolerance, 0.7 min). Missing values were filled using the software’s Fill Gap feature (mass tolerance, 5 ppm; signal/noise tolerance, 1.5). Compound identification was achieved by matching the mass and retention time of observed peaks to an in-house database generated using metabolite standards (mass tolerance, 5 ppm; RT tolerance, 0.5 min). Peak annotations were further confirmed using mzCloud (ddMS2) database search (precursor and fragment mass tolerance, 10 ppm; match factor threshold, 50). Within Compound Discoverer, data were normalized using a constant median strategy, and PCA was also performed using only features identified by the above strategy (in-house database and mzCloud). The weighted distance of in vitro conditions to tumor samples was calculated using the following equation

$$\text{Weighted distance}\;=\sqrt{{\left((\text{PC}1{\text{rep}}_{n}-\text{PC}1{\text{tum}}_{\text{mean}})\times 0.49\right)}^{2}+{\left((\text{PC}2{\text{rep}}_{n}-\text{PC}2{\text{tum}}_{\text{mean}})\times 0.218\right)}^{2}}$$

where PC1rep_*n*_ is the PC1 value of each in vitro replicate and PC1tum_mean_ is the mean PC1 value of all tumor samples.

## Supplementary Material

http://advances.sciencemag.org/cgi/content/full/5/1/eaau7314/DC1

## SUPPLEMENTARY MATERIALS

Supplementary material for this article is available at http://advances.sciencemag.org/cgi/content/full/5/1/eaau7314/DC1 Table S1. Comparison between the formulations of Plasmax and HPLM. Fig. S1. Selenite-dependent colony formation. Fig. S2. PCA of gene expression. Fig. S3. Isotopologue distribution of urea cycle intermediates.
