# Supplementary material for: Improving the metabolic fidelity of cancer models with a physiological cell culture medium
Source: Sci Adv. 2019 Jan 2;5(1):eaau7314. doi: 10.1126/sciadv.aau7314 (PMC6314821; doi:10.1126/sciadv.aau7314)
Supplement: http://advances.sciencemag.org/cgi/content/full/5/1/eaau7314/DC1 [file supp_5_1_eaau7314__index.html]

Science Advances | Science Advances

## Supplementary Materials

**This PDF file includes:**

- Table S1. Comparison between the formulations of Plasmax and HPLM.
- Fig. S1. Selenite-dependent colony formation.
- Fig. S2. PCA of gene expression.
- Fig. S3. Isotopologue distribution of urea cycle intermediates.

Download PDF

**Files in this Data Supplement:**

- Adobe PDF - aau7314\_SM.pdf
