# Supplementary material for: Improving the metabolic fidelity of cancer models with a physiological cell culture medium
Source: Sci Adv. 2019 Jan 2;5(1):eaau7314. doi: 10.1126/sciadv.aau7314 (PMC6314821; doi:10.1126/sciadv.aau7314)
Supplement: http://advances.sciencemag.org/cgi/content/full/5/1/eaau7314/DC1 [file aau7314_SM.pdf]

Science Advances
AAAS

advances.sciencemag.org/cgi/content/full/5/1/eaau7314/DC1

Supplementary Materials for

Improving the metabolic fidelity of cancer models with a physiological cell culture medium

Johan Vande Voorde, Tobias Ackermann, Nadja Pfetzer, David Sumpton, Gillian Mackay, Gabriela Kalna, Colin Nixon, Karen Blyth, Eyal Gottlieb, Saverio Tardito*

*Corresponding author. Email: s.tardito@beatson.gla.ac.uk

Published 2 January 2019, *Sci. Adv.* **5**, eaau7314 (2019)
DOI: 10.1126/sciadv.aau7314

This PDF file includes:

Table S1. Comparison between the formulations of Plasmax and HPLM.
Fig. S1. Selenite-dependent colony formation.
Fig. S2. PCA of gene expression.
Fig. S3. Isotopologue distribution of urea cycle intermediates.

SUPPLEMENTARY MATERIAL

Table S1. Comparison between the formulations of Plasmax and HPLM.

	Concentration (μM)	
	**Plasmax**	**HPLM**
**Proteinogenic Amino Acids**		
L-Alanine[1]	510	430
L-Arginine[1]	64	110
L-Asparagine[1]	41	50
L-Aspartic acid[1]	6	20
L-Cysteine[2]	33	40
L-Glutamate[1]	98	80
L-Glutamine[5]	650	550
Glycine[1]	330	300
L-Histidine[1]	120	110
L-Isoleucine[1]	140	70
L-Leucine[1]	170	160
L-Lysine[1]	220	200
L-Methionine[1]	30	30
L-Phenylalanine[1]	68	80
L-Proline[1]	360	200
L-Serine[1]	140	150
L-Threonine[1]	240	140
L-Tryptophan[1]	78	60
L-Tyrosine[1]	74	80
L-Valine[1]	230	220
**Non-proteinogenic Amino Acids**		
α–Aminobutyrate[2]	41	20
L-Citrulline[1]	55	40
L-Cystine[1]	65	100
L-Homocysteine[2]	9	NA
4-Hydroxy-L-proline[2]	13	20
L-Ornitine[1]	80	70
L-Pyroglutamate[2]	20	NA
**Amino Acids Derivatives**		
L-Acetyl glycine[2]	70	90
L-Carnosine[2]	6	NA
Glutathione (reduced)[2]	37	25
Taurine[2]	130	90
N-Trimethylglycine (betaine) [2]	72	70
**Other Components**		
Acetate[2]	42	40
Acetone[2]	55	60
Acetyl carnitine[2]	5	NA
Citrate[2]	114	130
Carnitine[2]	46	40
Creatine[2]	37	40
Creatinine[2]	74	75
Formate[2]	33	50
Fructose	NA	40
Galactose	NA	60
D-Glucose[7]	5560	5000
Glycerol[2]	82	120
2-Hydroxybutyrate[2]	31	50

3-Hydroxybutyrate[2]	77	50
3-Hydroxyisobutyrate[2]	20	NA
Hypoxanthine[2]	5	10
Lactate[2]	500	1600
Malonate	NA	10
Methyl acetoacetate[2]	41	NA
Phenol Red[7]	25	14
Pyruvate[8]	100	50
Succinate[2]	23	20
Uracil[2]	2	NA
Urate[4]	270	350
Urea[2]	3000	5000
Uridine[2]	3	NA
**Inorganic Salts**		
Ammonium Chloride[3]	50	40
Calcium Chloride[7]	1800	2350
Calcium Nitrate	NA	40
Magnesium Chloride	NA	480
Magnesium Sulfate[7]	813	350
Potassium Chloride[7]	5330	4100
Sodium Bicarbonate[7]	26191	24000
Sodium Chloride[7]	118706	105000
Sodium Phosphate monobasic[7]	1010	870*
**Trace Elements**		
Ammonium Metavanadate[3]	0.0026	NA
Cupric Sulfate[3]	0.0052	NA
Ferric Nitrate[3]	0.1238	NA
Ferric Sulfate[3]	1.0428	NA
Manganous Chloride[3]	0.0002	NA
Sodium Selenite[3]	0.0289	NA
Zinc Sulfate[3]	1.5	NA
**Vitamins**		
p-Aminobenzoate	NA	7.3
Ascorbate[6]	62	NA
D-Biotin[5]	4.1	0.8
Choline[5]	7.1	21.5
Folate[5]	2.3	2.3
myo-Inositol[5]	11.1	194.3
Niacinamide[5]	8.2	8.2
D-Pantothenic acid hemicalcium[5]	4.2	1.05
Pyridoxine[5]	4.9	4.9
Riboflavin[5]	0.3	0.5
Thiamine[5]	3	3
Vitamin B12[6]	0.005	0.0037

* present as dibasic salt

Formulations of Plasmax and HPLM (Cantor et al. 2017, Cell 169, 258-272). Plasmax components were dissolved and stocked as follow: [1] 100x solution 1, [2] 100x solution 2, [3] 1,000x solution 3 (trace element concentrations as in Advanced DMEM-F12, Thermo Fisher Scientific cat no. 12634028), [4] 500x solution 4, [5]100x commercially available BME vitamin mix. [6]100,000x individual stocks, supplemented to BME vitamin mix to obtain a 100x stock solution 5. [7] included in the commercially available EBSS. [8] individual stock solutions commercially available.

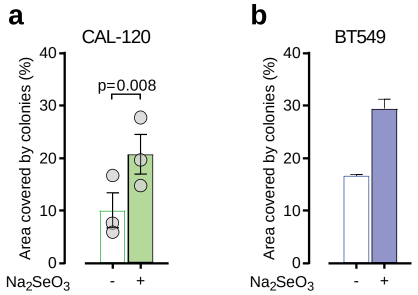

Fig. S1. Selenite-dependent colony formation. Quantification of colony formation assays performed with (a) CAL-120 cells and (b) BT549 seeded 500cells/well and incubated in DMEM-F12 with 28nM Na_2SeO_3, as indicated. Mean ± SEM; (a) n = 3 (b) n=1 independent experiments. Each dot represents an independent experiment. p value refers to a two-tailed t-test for paired homoscedastic samples.

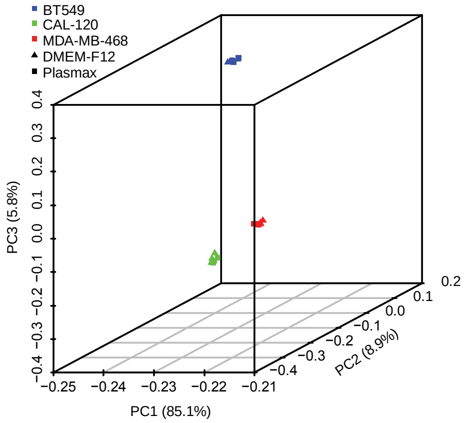

Fig. S2. PCA of gene expression obtained from RNA sequencing data of BT549, CAL-120, and MDA-MB-468 cells cultured in Plasmax or DMEM-F12, in normoxia. Each dot represents an independent experiment.

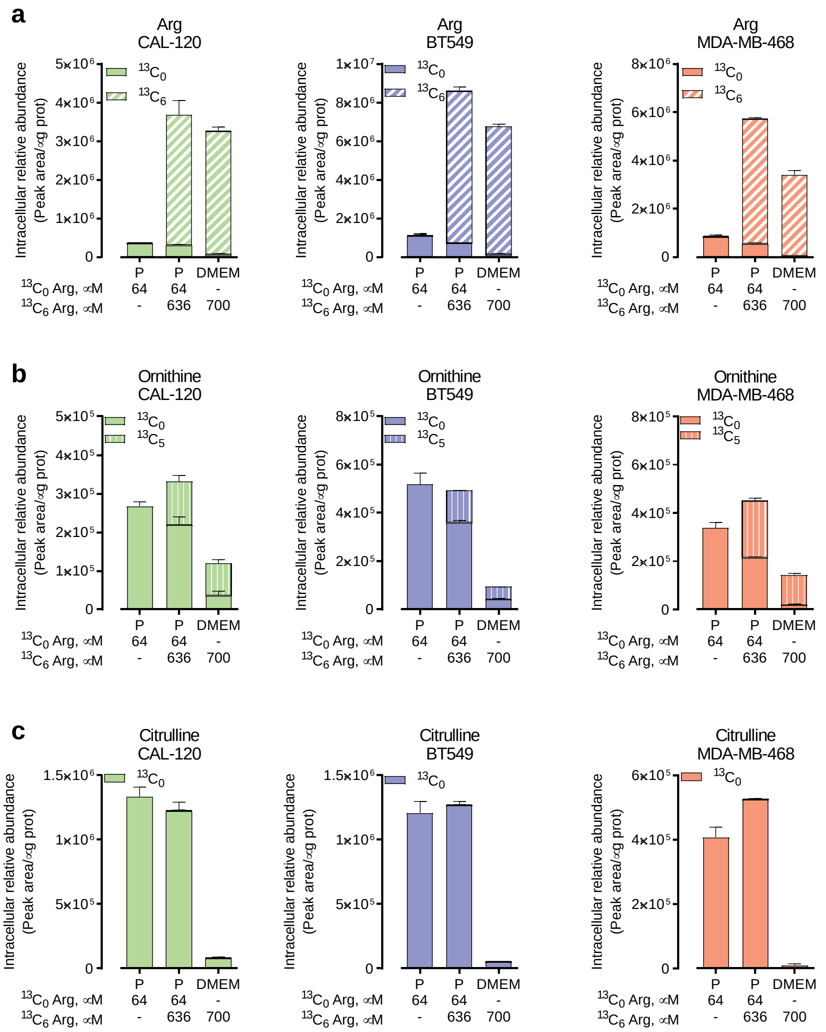

Fig. S3. Isotopologue distribution of urea cycle intermediates. Intracellular levels of (**a**) ^{13}C arginine (**b**) ^{13}C ornithine and (**c**) ^{13}C citrulline in BT549, CAL-120 and MDA-MB-468 cells cultured for 48 hours in Plasmax (P) and DMEM supplemented with $^{13}C_6$ and $^{13}C_0$ arginine at the indicated concentrations. Mean ± SEM; CAL-120 (n = 3 independent experiments); BT549 and MDA-MB-468 (n = 2 independent experiments).
